# Organizational justice, psychological distress, and stress-related behaviors by occupational class in female Japanese employees

**DOI:** 10.1371/journal.pone.0214393

**Published:** 2019-04-11

**Authors:** Yumiko Kobayashi, Naoki Kondo

**Affiliations:** Department of Health Education and Health Sociology, School of Public Health, The University of Tokyo, Tokyo, Japan; Medical University Hospital Tuebingen, GERMANY

## Abstract

**Backgrounds:**

Recent evidence has suggested that in Japan, professionals and managers have a higher risk of poor health than other workers (e.g., clerks and manual laborers), and this effect may be stronger among women than men. Low organizational justice, which is known to be a potential risk factor for poor health among employees, may explain the gender-specific association.

**Methods:**

We examined the associations between perceived organizational justice and psychological distress and stress-related behaviors (smoking and heavy drinking) in 2,216 female and 7,557 male employees aged 18 to 69 years from the Japanese Study of Health, Occupation, and Psychosocial Factors Related Equity. We measured both procedural and interactional justice, and compared managers and professionals with other employees.

**Results:**

After adjusting for demographic characteristics and occupational stress, low levels of perceived procedural and interactional justice were found to be associated with a high prevalence of psychological distress for both women and men, regardless of occupational status. Among female managers and professionals, perceived interactional justice (measured as the levels of supports by supervisors, etc.) was significantly associated with smoking, whereas no such association was observed among other workers. When interactional justice was perceived to be low, the prevalence of smoking was 6.5 percentage points higher among managers and professionals than among others. Neither procedural nor interactional justice was associated with risk of heavy drinking.

**Conclusions:**

Female managers and professionals in a workplace with unsupportive supervisors may be more likely to engage in unhealthy coping behaviors to manage their stress. Creating supportive workplaces may be beneficial in increasing workers’ health, especially for female managers and professionals.

## Introduction

Mental illness and psychological distress are key topics in industrial health. Research suggests that psychological distress attributable to job strain and effort-reward imbalances increases the worker’s risk of cardiovascular disease and psychological disorders [1,2]. In Japan, studies have suggested that lingering economic recessions since the 1980s and strong psychological distress may be associated with depression and suicide among male workers [3,4]. The potentially increasing distress among working women may be attributable to the recent increase in women’s labor market participation, although a wide gender gap in the workforce remains [5,6]. Umeda et al. found that Japanese female employees have low self-esteem in their jobs and perceive that they are isolated and that their work is less rewarded [6].

Among the workplace environmental factors, organizational justice has been actively studied as a determinant of health and of health inequality among workers, and we consider that to be an important factor explaining the potential gender gap in workplace psychosocial conditions [7]. Organizational justice refers to the extent to which employees are treated fairly in their workplace [8], and consists of procedural justice and interactional justice [9]. The former refers to whether decision-making processes include input from affected parties, are consistently applied, avoid bias, are correctable, are accurate, and are ethical [10]. The latter refers to whether employees are treated with consideration and courtesy by their supervisors [10]. Recent studies suggest a relationship between low perceived organizational justice and poor health, as measured by indicators such as high blood pressure, psychological distress, insomnia, and inflammatory markers [11–14].

Recent studies also suggest differing strengths of association between organizational justice and health according to socioeconomic status, with evidence of increased health risks among those who are worse off in public sector workplaces with poor procedural justice [10,11]. However, in private companies in Japan, increased physical and mental health risks were observed among managers rather than other employees, including clerical workers and manual laborers, despite the privileged occupational position of such employees [3,5,6]. As for gender differences, we thought that, in Japan’s current working contexts in private companies, workplace environments, as characterized by organizational injustice, may affect women in the management and professional positions in particular, because of their potential risks for isolation in the workplace. Statistics shows that the share of women in management and professional positions is low (less than 10% in the private sector), and the limited number of peers in the same positions in the workplace could lead to social isolation, depriving women of the opportunities of having necessary peer support to alleviate psychological distress [1,15].

To our knowledge, few studies have examined the gender-specific associations between organizational justice and psychosocial distress or relevant health behaviors, specifically focusing on their occupational statuses. In this study, we therefore investigated the relationships between organizational justice and risk of psychological distress and two stress-related behaviors–namely, smoking and heavy drinking–using data collected in multiple Japanese private companies. Given the recently-discovered evidence of poorer health among female managers and professionals than among other workers, the associations between organizational injustice and the stress-related outcomes might be stronger among women than men, as well as among managers and professionals compared to other workers [5,6].

## Methods

### Participants

In the present study, we used data collected in the first survey waves of the Study of Health, Occupation, and Psychosocial Factors Related Equity (J-HOPE), an occupational cohort study on social class and health conducted from 2010 to 2014 in Japan. This study was approved by the Ethics review Board of the University of Tokyo Faculty of Medicine., Kitasato University School of Medicine/Hospital, and University of Occupational and Environmental Health. The response rate was 77%. Data were mainly collected at health checkup opportunities; it is legally mandated that employers provide such opportunities for all employees. According to the survey on the health of workers in 2012, about 80% of workers were undergoing medical check-up [16]. A total of 14,189 employees from 12 companies (main industries: manufacturing, transportation, service industries, information technology, and hospitals/medical facilities) were surveyed using a questionnaire that was self-administered after consent was obtained. Excluding the data of those who had missing age information, the final number of female employees included in the present analysis was 2,216, and the final number of male employees was 7,557.

### Measures

#### Outcome variables

Psychological distress was evaluated using the Japanese version of the Kessler 6 (K6) scale [17]. The K6 scale consists of six items that measure the frequency of psychological distress during the previous 30 days: How often did you feel (1) nervous? (2) hopeless? (3) restless or fidgety? (4) so depressed that nothing could cheer you up? (5) that everything was an effort? (6) worthless? The response options ranged from 0 (*none of the time*) to 4 (*all of the time*), and the total score for each participant was derived by summing the item scores with equal weight. We categorized participants as either exhibiting psychosocial distress (scores ≧5) or not (scores <5), based on Sakurai et al.’s suggested cut-off [18]. Smoking status and frequency of alcohol drinking were self-reported using the following items: (1) Do you smoke? (2) Do you drink alcohol? The response options ranged from 1 (*never*) to 3 (*currently*) and from 1 (*no*) to 3 (*almost every day*), respectively. For the purpose of our study, we regarded those who reported smoking currently as smokers, and those who reported drinking alcohol almost every day as heavy drinkers.

#### Organizational justice

Organizational justice was measured using the Japanese version of the Organizational Justice Questionnaire (OJQ), which is a translation of the original English version [8]. The reliability and validity of the Japanese version have been evaluated [19]. The OJQ measures two components of organizational justice: procedural justice and interactional justice. The procedural justice subscale consists of the following seven items: (1) Decisions are made based on accurate information, (2) People are provided with opportunities to appeal or challenge decisions they find unsuccessful, (3) All sides affected by the decision are represented in decision-making, (4) Decisions are made with consistency (the rules are the same for every employee), (5) The concerns of all those affected by the decision are heard before decision-making, (6) Feedback is collected regarding the decision and its implementation, and (7) It is possible to request clarification or additional information about the decision. The interactional justice subscale consists of the following six items: (1) Our supervisor considers our viewpoint, (2) Our supervisor is able to suppress personal biases, (3) Our supervisor provides us with timely feedback about decisions and their implications, (4) Our supervisor treats us with kindness and consideration, (5) Our supervisor shows concern for our rights as employees, and (6) Our supervisor takes steps to deal with us in a truthful manner. All items of both subscales are rated on a 5-point Likert scale ranging from 1 (*strongly disagree*) to 5 (*strongly agree*). In this study, we calculated the sum of each participant’s responses to each subscale and categorized participants by tertiles, in the same way as previous studies [20,21].

#### Occupational positions and covariates

In the J-HOPE, occupational position was evaluated based on which set of duties were most similar to those carried out by the respondent, including managers, professionals, technicians, clerks, service and sales workers, craft and related trade workers, machine operators and assemblers, laborers, and others. We dichotomized occupational status into managers and professionals vs. others based on skill specialization and skill level.

We also entered educational attainment and household income into the analysis as covariates. We categorized educational attainment as (1) junior high school, (2) senior high school, or (3) college or higher. We categorized household income as (1) ≦ JPY 2.99 million, (2) JPY 3–4.99 million, (3) JPY 5–7.99 million, (4) JPY 8–9.99 million, (5) JPY 10–14.99 million, or (6) ≧ JPY 15 million. Other covariates included age and working hours per week. Working hours were classified as (1) ≦ 30 hr, (2) 31–40 hr, (3) 41–50 hr, (4) 51–60 hr, or (5) ≧ 61 hr. To assess other indicators of job stress, we used the 10-item Japanese version of the Effort-Reward Imbalance Questionnaire (ERIQ) and the 22-item Japanese version of the Job Content Questionnaire (JCQ) [1,2,22,23]. The ERIQ includes sub-scales for effort and reward, with each item rated on a 4-point scale. To measure the extent of the imbalance, we calculated the ratio between the scores for effort and reward; we then categorized participants as perceiving a high (>1.4) or low (≦1.4) ratio using a cut-off point based on a previous study [23]. The JCQ consists of subscales relating to job demands and job control, with each item rated on a 4-point scale. We collated participants’ responses to these items to produce a single index of job demands and control [22].

### Statistical analysis

After computing descriptive statistics for participants’ general characteristics, we calculated the prevalence ratios (PRs) of psychological distress (≧5), smoking, and heavy drinking, along with their 95% confidence intervals (CIs), between levels of perceived organizational justice, for workers of different occupational status [24]. The hypothetically most advantaged group (that of managers and professionals) was selected as the reference category. We used Poisson regression with robust standard errors. In multivariate models, we adjusted for age, levels of education, household income, and working hours per week. To evaluate the role of conditions relating to job stress, we further adjusted for effort-reward imbalance and JCQ score. We also examined the interactions between occupational status (managers and professionals vs. others) and type of organizational justice with three levels of each justice (high/intermediate/low).

For comparison, we also conducted the same analyses for males. Statistical analyses were conducted using STATA 11.1 for Windows.

## Results

The J-HOPE participants included in the present analyses consisted of 420 female managers and professionals, and 1,796 other female workers. Among these, 58.57% of managers and professionals and 52.39% of other workers exhibited psychological distress. The proportion of smokers in each group was 9.52% and 11.41%, respectively, and the proportion of heavy drinkers was 13.57% and 12.14%, respectively (Table 1).

**Table 1:** Baseline characteristics of employees, n (%) or mean [SD].

Variables		Women		Men	
		Managers & professionals (n = 420)	Other (n = 1796)	Managers & professionals(n = 2641)	Other (n = 4916)
**Age (years)**		35.39[9.03]	39.32[10.52]	43.69[9.13]	40.38[11.09]
**Educational level**	College or higher	394(93.81)	838(46.66)	2214(83.83)	2192(44.59)
	Senior High School	22(5.24)	898(50)	403(15.26)	2535(51.57)
	Junior High School	4(0.95)	48(2.67)	19(0.72)	159(3.23)
	Missing	0(0)	12(0.67)	5(0.19)	30(0.61)
**Household income**	≦ JPY 2.99 million	37(8.81)	405(22.55)	36(1.36)	340(6.92)
	JPY 3–4.99 million	114(27.14)	426(23.72)	247(9.35)	1189(24.19)
	JPY 5–7.99 million	122(29.05)	532(29.62)	730(27.64)	2288(46.54)
	JPY 8–9.99 million	48(11.43)	200(11.14)	714(27.04)	716(14.56)
	JPY 10–14.99 million	77(18.33)	156(8.69)	808(30.59)	326(6.63)
	≧JPY 15 million	22(5.24)	33(1.84)	100(3.79)	40(0.81)
	Missing	0(0)	44(2.45)	6(0.23)	17(0.35)
**Working hours per week**	30hr or less	7(1.67)	563(31.35)	60(2.27)	155(3.15)
	31-40hr	106(25.24)	762(42.43)	377(14.27)	1642(33.4)
	41-50hr	225(53.57)	366(20.38)	1396(52.86)	2278(46.34)
	51-60hr	63(15)	65(3.62)	612(23.17)	632(12.86)
	≧61hr	18(4.29)	23(1.28)	190(7.19)	180(3.66)
	Missing	1(0.24)	17(0.95)	6(0.23)	29(0.59)
**Procedural justice**	High	120(28.57)	521(29.01)	1157(43.81)	1659(33.75)
	Intermediate	142(33.81)	681(37.92)	852(32.26)	1695(34.48)
	Low	158(37.62)	594(33.07)	632(23.93)	1562(31.77)
**Interactional justice**	High	154(36.67)	589(32.8)	1209(45.78)	1726(35.11)
	Intermediate	127(30.24)	532(29.62)	814(30.82)	1400(28.48)
	Low	139(33.1)	675(37.58)	618(23.40)	1790(36.41)
**Effort-reward Imbalance**	≧1.4	338(80.48)	1571(87.47)	2298(87.01)	4173(84.89)
	< 1.4	78(18.57)	155(8.63)	286(10.83)	602(12.25)
	Missing	4(0.95)	70(3.9)	57(2.16)	141(2.87)
**Job demands**	High	109(25.95)	837(46.6)	706(26.73)	1610(32.75)
	Intermediate	121(28.81)	503(28.01)	881(33.36)	1667(33.91)
	Low	190(45.24)	435(24.22)	1049(39.72)	1616(32.87)
	Missing	0(0)	21(1.17)	5(0.19)	23(0.47)
**Job control**	High	195(46.43)	328(18.26)	1649(62.44)	1929(39.24)
	Intermediate	113(26.9)	399(22.22)	588(22.26)	1283(26.10)
	Low	110(26.19)	1036(57.68)	393(14.88)	1657(33.71)
	Missing	2(0.48)	33(1.84)	11(0.42)	47(0.96)
**Psychological distress (K6 score)**	< 5	174(41.43)	855(47.61)	1405(53.20)	2320(47.19)
	≧ 5	246(58.57)	941(52.39)	1236(46.80)	2596(52.81)
**Frequency of alcohol drinking**	None	175(41.67)	1010(56.24)	595(22.53)	1661(33.79)
	Sometimes	188(44.76)	568(31.63)	1013(38.36)	1660(33.77)
	Everyday	57(13.57)	218(12.14)	1033(39.11)	1595(32.45)
**Smoking status**	Never	358(85.24)	1514(84.3)	1425(53.96)	2410(49.02)
	Former	22(5.24)	77(4.29)	408(15.45)	661(13.45)
	Current	40(9.52)	205(11.41)	808(30.59)	1845(37.53)

Among men, 2,641 managers and professionals, and 4,916 other workers responded. Of these, 46.80% of managers and professionals and 52.81% of other workers exhibited psychological distress. The proportion of smokers in each group was 30.59% and 37.53%, respectively, and the proportion of heavy drinkers was 39.11% and 32.45%, respectively (Table 1).

**Fig 1:**
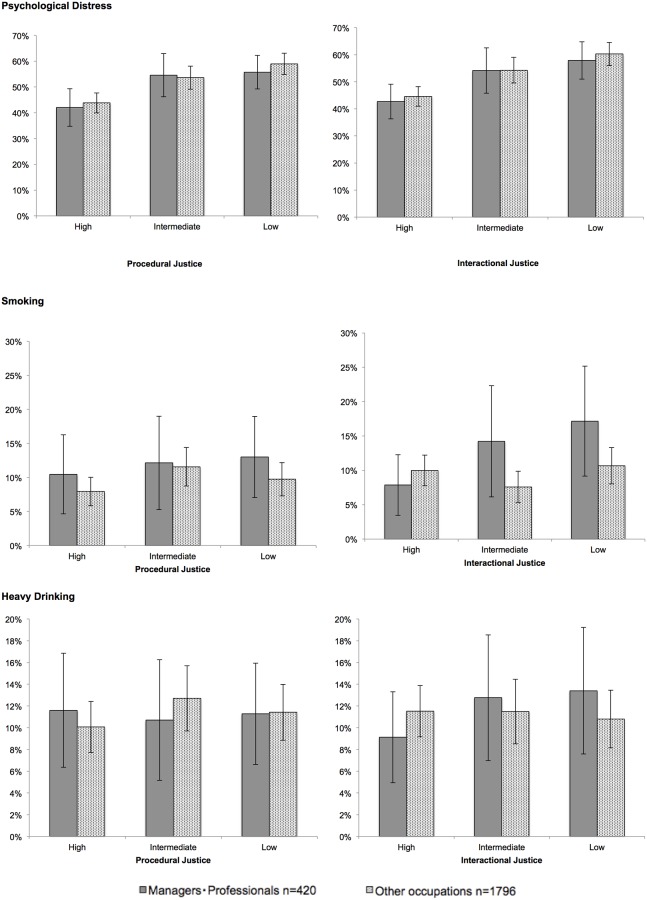
Prevalence of psychological distress, smoking, and drinking among female by organizational justice levels and occupation. Adjusted for age, education, household income, working hours, Effort-Reward Imbalance, Job demands and Job control. Error bars = 95% Confidence Intervals.

Among women, the prevalence of psychological distress among those reporting lower perceived levels of organizational justice was significantly higher among both managers and professionals and other workers. Compared to high procedural justice, the raw PR was 1.55 (95% CI: 1.25–1.91) among managers and professionals, and 1.43 (95% CI: 1.18–1.73) among other workers (Table 2). The corresponding PRs were 1.59 (95% CI: 1.33–1.90) and 1.33 (95% CI: 1.13–1.56) for low interactional justice. These associations remained statistically significant even after adjusting for multiple covariates. As shown in Fig 1, the multivariate-adjusted prevalence gap between workers perceiving high and low levels of organizational justice was nearly 20 percent points, regardless of occupational status and the type of organizational justice in question (Fig 1). Among men, we found similar occupation-related gradients in psychological distress (low levels of procedural justice exhibited an adjusted PR of 1.73 (95% CI: 1.58–1.89) among managers and professionals and 1.56 (95% CI 1.43–1.69) among other workers, and low levels of interactional justice were 1.70 (95% CI: 1.56–1.85) and 1.60 (95% CI: 1.48–1.73), respectively), whereas no gradients were found in smoking and heavy alcohol drinking.

**Table 2:** Prevalence ratios and 95% confidence intervals (CI) of psychological distress by organizational justice.

		Model 1		Model 2		Model 3	
**Women**	**Procedural justice**						
Managers & professionals (n = 420)	High	1.00 (ref)		1.00 (ref)		1.00 (ref)	
	Middle	1.44(1.14–1.80)	<0.001	1.40(1.12–1.76)	<0.001	1.30(1.04–1.62)	0.02
	Low	1.55(1.25–1.91)	<0.001	1.55(1.26–1.90)	<0.001	1.33(1.09–1.62)	0.01
Other (n = 1796)	High	0.99(0.81–1.21)	0.89	1.05(0.86–1.28)	0.67	1.04(0.86–1.27)	0.68
	Middle	1.16(0.95–1.42)	0.14	1.29(1.06–1.58)	0.01	1.28(1.05–1.55)	0.01
	Low	1.43(1.18–1.73)	<0.001	1.56(1.29–1.89)	<0.001	1.40(1.16–1.69)	<0.001
**Men**	**Procedural justice**						
Managers & professionals (n = 2641)	High	1.00 (ref)		1.00 (ref)		1.00 (ref)	
	Middle	1.39(1.26–1.55)	<0.001	1.37(1.24–1.52)	<0.001	1.28(1.15–1.41)	<0.001
	Low	1.77(1.62–1.94)	<0.001	1.73(1.58–1.89)	<0.001	1.46(1.34–1.60)	<0.001
Other occupations (n = 4916)	High	1.21(1.11–1.32)	<0.001	1.12(1.02–1.22)	0.02	1.11(1.01–1.21)	0.03
	Middle	1.52(1.40–1.65)	<0.001	1.41(1.29–1.54)	<0.001	1.28(1.17–1.40)	<0.001
	Low	1.68(1.55–1.82)	<0.001	1.56(1.43–1.69)	<0.001	1.32(1.21–1.44)	<0.001
**Women**	**Interactional justice**						
Managers & professionals (n = 420)	High	1.00 (ref)		1.00 (ref)		1.00 (ref)	
	Middle	1.30(1.05–1.60)	0.02	1.35(1.09–1.67)	0.01	1.27(1.03–1.56)	0.03
	Low	1.59(1.33–1.90)	<0.001	1.55(1.29–1.86)	<0.001	1.36(1.13–1.62)	<0.001
Other (n = 1796)	High	0.94(0.80–1.12)	0.49	1.03(0.87–1.22)	0.76	1.04(0.88–1.24)	0.62
	Middle	1.12(0.94–1.33)	0.20	1.29(1.08–1.53)	0.01	1.27(1.07–1.51)	0.01
	Low	1.33(1.13–1.56)	<0.001	1.54(1.30–1.82)	<0.001	1.41(1.19–1.67)	<0.001
**Men**	**Interactional justice**						
Managers & professionals (n = 2641)	High	1.00 (ref)		1.00 (ref)		1.00 (ref)	
	Middle	1.31(1.19–1.45)	<0.001	1.31(1.19–1.45)	<0.001	1.22(1.11–1.35)	<0.001
	Low	1.72(1.58–1.88)	<0.001	1.70(1.56–1.85)	<0.001	1.45(1.33–1.59)	<0.001
Other occupations (n = 4916)	High	1.15(1.06–1.25)	<0.001	1.07(0.99–1.17)	0.09	1.06(0.98–1.15)	0.15
	Middle	1.38(1.27–1.50)	<0.001	1.31(1.20–1.43)	<0.001	1.22(1.11–1.33)	<0.001
	Low	1.66(1.55–1.79)	<0.001	1.60(1.48–1.73)	<0.001	1.35(1.25–1.47)	<0.001

Model 1 Unadjusted

Model 2 Adjusted for age, education level, household income, and working hours per week.

Model 3 Additionally adjusted for effort-reward imbalance and job control and job demand.

A prevalence gap for smoking was observed only in relation to interactional justice among women (Table 3). Even adjusting for multiple covariates, compared to managerial and professional workers reporting high levels of interactional justice, those reporting low levels of interactional justice exhibited an adjusted PR of 2.18 (95% CI: 1.08–4.40) among managers and professionals and 1.36 (95% CI 0.72–2.56) among other workers (Table 3). Among those who reported high levels of interactional justice, the prevalence of smoking was lower among managers and professionals than among others (7.9% vs. 10.0%), whereas this was reversed, and the difference was statistically significant, among those reporting lower interactional justice (17.2% vs. 10.7%; for the interaction, *p* = 0.04 for intermediate and 0.06 for low interactional justice). Procedural justice and interactional justice were not related to differences in alcohol intake among participants in any occupation (Table 4).

**Table 3:** Prevalence ratios and 95% confidence intervals (CI) of smoking by levels of organizational justice.

		Model 1		Model 2		Model 3	
**Women**	**Procedural justice**						
Managers & professionals (n = 420)	High	1.00 (ref)		1.00 (ref)		1.00 (ref)	
	Middle	1.17(0.54–2.56)	0.69	1.19(0.55–2.58)	0.67	1.16(0.53–2.53)	0.71
	Low	1.32(0.65–2.67)	0.44	1.36(0.68–2.75)	0.39	1.25(0.62–2.49)	0.54
Other occupations (n = 1796)	High	1.21(0.67–2.17)	0.54	0.83(0.45–1.52)	0.55	0.76(0.41–1.40)	0.38
	Middle	1.63(0.91–2.92)	0.10	1.12(0.61–2.06)	0.72	1.11(0.60–2.05)	0.75
	Low	1.40(0.78–2.52)	0.26	1.01(0.55–1.86)	0.98	0.93(0.50–1.74)	0.82
**Men**	**Procedural justice**						
Managers & professionals (n = 2641)	High	1.00 (ref)		1.00 (ref)		1.00 (ref)	
	Middle	1.07(0.94–1.23)	0.31	1.05(0.92–1.21)	0.45	1.05(0.92–1.21)	0.46
	Low	0.97(0.84–1.12)	0.64	0.98(0.85–1.14)	0.82	0.99(0.85–1.15)	0.87
Other occupations (n = 4916)	High	1.24(1.13–1.37)	<0.001	1.03(0.93–1.15)	0.57	1.04(0.93–1.16)	0.48
	Middle	1.29(1.16–1.43)	<0.001	1.06(0.95–1.18)	0.32	1.06(0.95–1.19)	0.30
	Low	1.19(1.07–1.32)	<0.001	0.99(0.88–1.10)	0.80	1.00(0.89–1.12)	0.98
**Women**	**Interactional justice**						
Managers & professionals (n = 420)	High	1.00 (ref)		1.00 (ref)		1.00 (ref)	
	Middle	1.75(0.80–3.84)	0.16	1.78(0.82–3.91)	0.15	1.81(0.83–3.95)	0.14
	Low	2.33(1.15–4.70)	0.02	2.27(1.13–4.57)	0.02	2.18(1.08–4.40)	0.03
Other occupations (n = 1796)	High	1.84(1.03–3.30)	0.04	1.32(0.71–2.42)	0.38	1.27(0.69–2.35)	0.44
	Middle	1.51(0.81–2.80)	0.20	0.96(0.50–1.83)	0.90	0.97(0.51–1.84)	0.91
	Low	2.22(1.23–3.99)	0.01	1.43(0.77–2.67)	0.26	1.36(0.72–2.56)	0.34
**Men**	**Interactional justice**						
Managers & professionals (n = 2641)	High	1.00 (ref)		1.00 (ref)		1.00 (ref)	
	Middle	1.12(0.97–1.28)	0.12	1.08(0.94–1.24)	0.28	1.08(0.94–1.24)	0.29
	Low	1.10(0.95–1.27)	0.22	1.04(0.90–1.21)	0.58	1.05(0.90–1.22)	0.53
Other occupations (n = 4916)	High	1.23(1.11–1.35)	<0.001	1.03(0.93–1.15)	0.52	1.05(0.94–1.16)	0.42
	Middle	1.31(1.18–1.46)	<0.001	1.06(0.94–1.18)	0.35	1.07(0.95–1.20)	0.28
	Low	1.34(1.21–1.48)	<0.001	1.04(0.93–1.16)	0.49	1.05(0.93–1.18)	0.41

Model 1 Unadjusted.

Model 2 Adjusted for age, education level, household income, and working hours per week.

Model 3 Additionally adjusted for effort-reward imbalance and job control and job demand.

**Table 4:** Prevalence ratios and 95% confidence intervals (CI) of heavy alcohol drinking by levels of organizational justice.

		Model 1		Model 2		Model 3	
**Women**	**Procedural justice**						
Managers・Professionals (n = 420)	High	1.00 (ref)		1.00 (ref)		1.00 (ref)	
	Middle	0.79(0.41–1.51)	0.48	0.83(0.44–1.58)	0.57	0.92(0.48–1.78)	0.81
	Low	1.02(0.59–1.76)	0.95	1.05(0.61–1.81)	0.87	0.97(0.55–1.73)	0.93
Other occupations (n = 1796)	High	0.76(0.49–1.20)	0.24	0.85(0.53–1.36)	0.50	0.87(0.52–1.44)	0.58
	Middle	0.92(0.59–1.44)	0.71	1.01(0.63–1.62)	0.98	1.10(0.66–1.82)	0.73
	Low	0.88(0.56–1.39)	0.59	0.93(0.58–1.49)	0.77	0.98(0.59–1.63)	0.95
**Men**	**Procedural justice**						
Managers & professionals (n = 2641)	High	1.00 (ref)		1.00 (ref)		1.00 (ref)	
	Middle	0.93(0.82–1.05)	0.22	0.97(0.87–1.09)	0.63	1.00(0.89–1.12)	0.95
	Low	0.88(0.78–0.99)	0.03	0.96(0.85–1.08)	0.47	1.00(0.88–1.13)	0.95
Other occupations (n = 4916)	High	0.82(0.76–0.90)	<0.001	0.99(0.9–1.09)	0.80	1.00(0.91–1.11)	0.92
	Middle	0.73(0.66–0.81)	<0.001	0.88(0.79–0.99)	0.03	0.90(0.81–1.01)	0.08
	Low	0.80(0.72–0.88)	<0.001	0.94(0.85–1.04)	0.23	1.00(0.89–1.11)	0.93
**Women**	**Interactional justice**						
Managers & professionals (n = 420)	High	1.00 (ref)		1.00 (ref)		1.00 (ref)	
	Middle	1.91(1.06–3.45)	0.03	1.67(0.93–2.98)	0.09	1.40(0.76–2.56)	0.28
	Low	1.64(0.91–2.97)	0.10	1.61(0.90–2.87)	0.11	1.47(0.81–2.66)	0.20
Other (n = 1796)	High	1.24(0.78–1.99)	0.36	1.29(0.79–2.11)	0.31	1.26(0.77–2.07)	0.36
	Middle	1.27(0.77–2.08)	0.35	1.26(0.74–2.12)	0.39	1.26(0.74–2.15)	0.40
	Low	1.27(0.79–2.06)	0.33	1.24(0.74–2.09)	0.41	1.18(0.70–2.01)	0.53
**Men**	**Interactional justice**						
Managers & professionals (n = 2641)	High	1.00 (ref)		1.00 (ref)		1.00 (ref)	
	Middle	1.11(0.99–1.24)	0.08	1.07(0.96–1.19)	0.24	1.06(0.94–1.18)	0.33
	Low	1.05(0.93–1.19)	0.45	1.05(0.93–1.19)	0.40	1.08(0.96–1.23)	0.21
Other occupations (n = 4916)	High	0.82(0.75–0.89)	<0.001	0.99(0.90–1.09)	0.89	1.00(0.91–1.10)	0.98
	Middle	0.89(0.80–0.99)	0.03	1.00(0.90–1.11)	0.99	1.01(0.91–1.13)	0.79
	Low	0.89(0.81–0.98)	0.02	0.97(0.87–1.07)	0.51	1.02(0.91–1.14)	0.76

Model 1 Unadjusted.

Model 2 Adjusted for age, education level, household income, and working hours per week.

Model 3 Additionally adjusted for effort-reward imbalance and job control and job demand.

## Discussion

The key findings of this study were that (1) the prevalence of psychological distress was higher among working women and men with low perceived levels of organizational justice, regardless of their occupational positions; and (2) among women low levels of perceived interactional justice were associated with twice the prevalence of smoking, compared with high levels of perceived interactional justice, among managers and professionals, while this association was weaker among other workers. These associations remained statistically significant even adjusting for variables relating to workplace psychosocial status, including job control/demands and effort-reward imbalance. In both women and men, poor perceived procedural justice was associated with psychological distress [10].

Our finding of an association between poor organizational justice and health is concordant with the findings of earlier studies in other countries [10,11,25]. Recent research targeting civil servants in Finland suggests that low levels of interactional justice are associated with a high prevalence of smoking, although differences between occupations were not evaluated [26]. Organizational justice might be an important environmental factor in mental health regardless of the structure of the workplace, labor system, and workplace culture. In Japan, a previous study has found high job strain to be significantly associated with an increased risk of stroke in a population of Japanese workers, especially among female managers [4]. The present study adds evidence that interactional justice or support from supervisors may be more important to female Japanese managers and professionals than to other workers.

This may in part be attributable to the male-dominant structure of the labor market in Japan, which results in a very small proportion of women in managerial positions in the private sector (10% or less) [15]. Being a member of a minority in the workplace could be harmful to a worker’s mental health in two ways. First, it may be hard for such workers to find a suitable mentor to consult and from whom to obtain emotional and instrumental support. This effect could be exacerbated if supervisors are not supportive, as they may even become another source of stress. In Japan, as many as 58.2% of managers take no action after receiving abuse from a superior, according to a governmental survey on the conditions under which workplace abuse occurs [27]. A lack of supportive colleagues could be more problematic for women: a recent study finds that women are more likely than men to employ coping behaviors involving verbal expression [28]. Second, female managers and professionals could be the victims of severe physical and mental stress as a result of the greater effort they must exert to maintain their job status in the workplace. This is a similar theory to John-Henryism, which posits a related explanation for the health-harming struggles of African Americans in the United States [29].

Although our findings may be explained by the effects of excessive work-related psychosocial stress, in our analysis the associations between organizational justice and psychological distress and smoking were independent of other work-related psychosocial characteristics (as measured by the JCQ and ERIQ) [30]. Thus, other explanations, such as work-life imbalances, may also explain our findings [31]. The traditional family norm of the male breadwinner still plays a major role in many families, meaning that women more often take on multiple unpaid roles in the home and in their communities [32]. Nationally-representative data suggest that approximately four more hours per week are consumed by housework and childcare for women than for men [33]. Therefore, full-time female workers are more likely to have multiple roles across the workplace, home, and community, which may sometimes be overwhelming, leading to poor health and unhealthy coping behaviors [34].

This study has several limitations. First, we used a self-reported measure of perceived organizational justice. An individual’s perceptions of their workplace could be influenced by personality and how they tend to appraise stress, factors that were not accounted for in this study. Second, all variables were measured by self-report, potentially producing a common method bias. Moreover, some measurements might not be valid enough to capture the problematic health behavior. For example, we did not find a difference in alcohol consumption by occupational class and organizational justice. This might be because frequency of drinking might not be sensitive enough to capture problematic alcohol consumption. Further research should evaluate workplace organizational justice, alcohol consumption, and other variables using more objective and accurate measures. Third, the participants in this study were employed at only 12 companies in Japan. Moreover, there are disparities in social security/welfare services and working conditions between full- and part-time workers. Hence, generalization of the findings of this study could be limited to full-time workers working for companies evaluated in this study. Nonetheless, the 12 companies covered various industries, including manufacturing, transportation, service industries, and others. Fourth, there may be additional unmeasured potential confounders in the relationships investigated, including work-family conflict, marital status, and family composition [31]. Fifth, the definition of occupational status in this study might differ from that employed in other studies [5,6].

## Conclusions

In conclusion, our findings provide evidence that organizational justice is important to build workplaces that support good mental health for all female workers in Japan, and that good support and understanding from one’s supervisor may be more important for female managers and professionals. As the Japanese government has stressed, organizational justice may be a key factor in achieving gender equity in the workforce [7,35,36]. A fair human resources management system and a fair performance evaluation system contribute to the social progress of women, which is emphasized in Japan’s governmental survey report aiming to achieve gender-equal workplaces (the Basic Survey of Gender Equality in Employment Management) [37]. Organizational justice is important not only in expanding opportunities for women, but also for the benefit for all employees’ health.
